# Higher risk of tuberculosis in combination therapy for inflammatory bowel disease: A nationwide population-based cohort study in South Korea

**DOI:** 10.1097/MD.0000000000022897

**Published:** 2020-10-30

**Authors:** Seong Ji Choi, Min Sun Kim, Eun Sun Kim, Juneyoung Lee, Jae Min Lee, Hyuk Soon Choi, Bora Keum, Yoon Tae Jeen, Hong Sik Lee, Hoon Jai Chun, Chang Duck Kim

**Affiliations:** a Division of Gastroenterology and Hepatology, Department of Internal Medicine, Korea University College of Medicine; b Department of Internal Medicine, Hanyang University College of Medicine; c Department of Biostatistics, Korea University College of Medicine, Seoul, Republic of Korea.

**Keywords:** azathioprine, inflammatory bowel disease, infliximab, TNF inhibitor, tuberculosis

## Abstract

Inflammatory bowel disease (IBD) in Asia has become increasingly prevalent. As a treatment of IBD, many immunomodulators and biological agents were introduced and shown to be effective in inducing and maintaining remission. However, many cases with treatment failure were reported. To overcome the failure, combination therapy of immunomodulatory and biologics have emerged, showing better outcomes by optimizing biologic pharmacokinetics and minimizing immunogenicity. Adversely, rates of tuberculosis (TB) have been increased as a result. The aim of this study is to compare the risk of TB according to the therapy using large population data.

We used data from the South Korean Health Insurance and Review Agency over the period 2008–2016 and calculated the hazard ratio (HR) for TB in IBD. We compared the risk of TB according to the medication: infliximab only, azathioprine only (AZA), combination of azathioprine and infliximab (CAI), azathioprine monotherapy and infliximab monotherapy (AIM), and azathioprine and infliximab whether simultaneously or separately (AISS).

In IBD patients, a total of 249 patients were identified as active TB. After one-to-one matching with age, sex and disease duration, the risks of TB were significantly higher in AZA group (HR, 2.06; 95% CI, 1.35–3.12, *P* < .001), AIM group (HR, 3.26; 95% CI, 1.18–9.05, *P* = .02), AISS group (HR, 3.50; 95% CI, 1.92–6.37, *P* < .001), and CAI group (HR, 5.67; 95% CI, 2.42–10.21, *P* < .001), and the HR increased gradually in this order. In UC patients, the results were in similar pattern, but this pattern was not observed in CD patients in our study.

Our study shows that Korean IBD patients are at risk of TB, and the risk increases with usage of IBD medication; moreover, the risk is the highest if combination therapy is used. These results highlight the importance of screening for TB in IBD patients, especially in combination therapy.

## 1 Introduction

Inflammatory bowel disease (IBD), comprising ulcerative colitis (UC) and Crohn's disease (CD), is an immune-mediated disorder that is characterized by chronic and relapsing-remitting inflammation of the gastrointestinal tract. Owing to its frequent relapse, induction and maintenance of remission are important in a treatment strategy. Although its precise etiology is still unknown, it is widely accepted that IBD is caused by complex interactions between genetic susceptibility, environment factors, and intestinal microbiota.^[1,2]^ There is no curative therapy for IBD because of its unknown etiology; however, the aim of therapy is to control the disease and maintain normal bowel physiology without complications.^[3]^ To modulate these interactions and immune responses, conventional therapies such as 5-ASA, antibiotics, corticosteroids, and immunomodulators have been widely used; however, these strategies could not modify the course of the disease or reduce its complications.^[4]^ During the identification of the disease entity of IBD, increased concentration of pro-inflammatory cytokine known as tumor necrosis factor (TNF) in the mucosa of IBD patients was found, and drugs targeting monoclonal antibodies that inhibit the effects of TNF began to be used frequently in moderate to severe disease.^[5]^

However, treatment failures, such as primary nonresponse or secondary loss of response, of anti-TNF inhibitors were reported to be 13%–46% of patients, and combination therapy with anti-TNF agent and immunomodulatory drug has been suggested as an alternative.^[6,7]^ The combination therapy of azathioprine and infliximab was first studied during clinical trials of anti-TNF agents, and several studies have showed that azathioprine optimized biologic pharmacokinetics and minimized immunogenicity of infliximab.^[8,9]^ These effects of combination therapy resulted in better clinical response than monotherapy with either drug in mucosal healing and suppression of antibody formation to anti-TNF.^[8,10,11]^

Despite these advantages, there is a continuing question as to the safety of combination therapy because of the combined use of two immunosuppressant medications. Furthermore, there is a controversy over the safety profile of combination therapy to the extent that some studies suggest a higher risk of opportunistic infections while some studies, to the contrary, suggest much less serious infections in combination therapy.^[8,12,13]^ Combination therapy in many randomized controlled trials showed higher risk of tuberculosis (TB), one of most common opportunistic infections; however, their results are limited to small sample sizes and/or a short study duration.^[14]^ Population-based studies are needed to make unbiased estimates of the long-term complications of combination therapy on IBD patients. In this study, we conducted a nationwide, population-based study using database collected between 2008 and 2016 to investigate the incidence and risk of TB in South Korean IBD patients, depending on the medication: azathioprine alone, infliximab alone, or in combination of these drugs.

## 2 Materials and methods

### 2.1 Data source

The South Korean government runs a mandatory nationwide insurance system (National Health Insurance; NHI), and all healthcare utilization information is registered in a comprehensive claim and reimbursement database operated by a quasi-public organization called Health Insurance and Review Agency (HIRA). This study used HIRA data from the NHI, which provides a comprehensive coverage of all medical services, including outpatient and inpatient care, and pharmacy costs, to more than 52 million people. Medical institutions submit the healthcare utilization information to NHI for reimbursement purposes, and these data are stored in HIRA after review and reimbursement. The NHI has several special systems and programs for efficient disease management and control as well as distribution of limited finances. The NHI launched the Rare Intractable Disease (RID) program in 2006 for certain rare diseases including IBD, and the diagnostic criteria for these disease are strict and uniform based on the NHI standards. The South Korea government also runs a copayment decreasing system (CDS) for 1600 different disease codes by assigning an additional V code, and the program provides much-needed financial aid to the enrolled patients. All diseases in the RID program are included in CDS along with other severe and significant diseases such as malignancies and TB. Because the collected data are reviewed before the reimbursement, diagnosis of diseases included in RID and/or CDS are reliable.

The HIRA database contains information on all patients including demographic characteristics, hospitalization and outpatient care history, principal diagnosis, and comorbidity using the International Classification of Disease 10th revision (ICD-10), prescription, and diagnostic and surgical procedures. The database for this study comprises all HIRA reimbursement data between 2007 and 2016. The data had been stripped of identifiers to ensure patient confidentiality. The study was approved by the Korea University Anam Hospital Institutional Review Board (ED17034).

### 2.2 Identification of IBD patients

We first identified the incidence of IBD and baseline characteristics of IBD patients. To identify IBD patients more accurately, we enrolled the patients with both ICD-10 code and V copayment code for IBD. For UC, those codes are K51 and V131, respectively, and for CD, they are K50 and V130, respectively. To qualify for the RID program and be assigned a V code, IBD patients must meet certain strict criteria.^[15]^ For UC, the patients must have 1) clinical manifestation with bloody diarrhea, rectal bleeding, urgency, and/or pus in stool for longer than 4 weeks, 2) endoscopic features with loss of vascularity, mucosal friability, erythema, spontaneous bleeding, and/or ulceration of colorectal mucosa in continuous and circumferential pattern, and 3) pathologic findings with diffuse inflammation in mucosa, lymphoplasmacytic lamina propria infiltrate, crypt architecture distortion, and/or ulcers. For CD, the patients must have 1) clinical manifestation with abdominal pain, diarrhea, weight loss, fever, malaise and/or extraintestinal inflammatory symptoms for longer than 6 weeks, 2) endoscopic features with longitudinal ulcer, cobblestone mucosal appearance, skip lesion, and/or perianal disease, and 3) pathologic findings showing a collection of epithelioid histiocytes, noncaseating granuloma, crypt distortion, patchy transmural inflammation, and/or ulcers.

### 2.3 Identification of newly diagnosed TB in IBD patients

**Figure 1:**
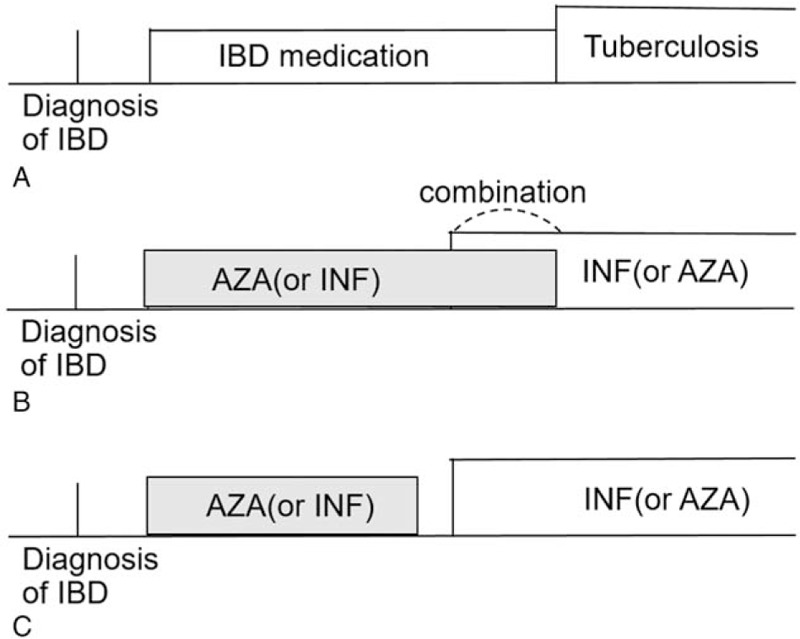
Definition of patients and drug regimen. (A) We selected our case patients to be diagnosed of TB after taking IBD medications. (B) We defined the combination therapy if AZA and INF were taken at the same time, CAI group, or (C) at different times, AIM group. AIM = azathioprine monotherapy and infliximab monotherapy, AZA = azathioprine only, CAI = combination of azathioprine and infliximab, IBD = inflammatory bowel disease, IFX, infliximab only.

To evaluate the risk of TB, it is important to identify newly diagnosed active TB patients and exclude old, indolent, and latent TB patients. Because South Korea is a high TB-incidence country, it is designated by law to report all new TB cases to nearby public health centers with a diagnosis code, hence the number of diagnosis codes and incidences of actual TB are almost identical. Because TB is covered financially by the CDS using V code, we set a patient's condition to have both ICD-10 code (A15-18, U84.3, U88) and V code (V206, V231, V246) for the diagnosis of TB. To select active TB cases and remove indolent and latent TB cases, we only included those cases that had been newly diagnosed as TB and had undergone TB treatments for at least 6 months with three or more anti-tuberculosis (anti-TB) drugs. Anti-TB drugs are isoniazid, rifampin, ethambutol and pyrazinamide, and other anti-TB drugs were not included for the purpose of excluding patients with reactivation TB or patients under chemoprophylaxis. Finally, to evaluate the effects of IBD itself and its medication in TB incidence, we included TB cases that had occurred after the start of IBD medication (Fig. 1A).

### 2.4 IBD medications

IBD medications reviewed in our study are as follows: 5-aminosalicylic acid (including sulfasalazine and mesalazine), corticosteroids (including oral and intravenous drugs), immunomodulators (including azathioprine and methotrexate), and anti-TNF drug (including infliximab and adalimumab). To evaluate the long-term risk of TB from these drugs and their combinations, we decided to use the drug combination of thiopurines and anti-TNF biologics, and among them, azathioprine and infliximab, respectively, are most commonly used. We compared the risk of TB according to the medication, which was divided into six groups: without immunomodulator (WI), infliximab only (IFX), azathioprine only (AZA), combination of azathioprine and infliximab (CAI), azathioprine monotherapy and infliximab monotherapy (AIM), and azathioprine and infliximab whether simultaneously or separately (AISS). The AIM group included only those patients who received both azathioprine therapy and infliximab therapy in different periods, and the CAI group only included those patients who received both azathioprine and infliximab in the same period. Figure 1B shows the results for CAI, and Figure 1C shows that for AIM. AISS group included all patients who underwent azathioprine and infliximab therapy, and we remark that AISS denotes the sum of CAI and AIM.

### 2.5 Statistical analysis

Data are presented as mean ± standard deviation (SD) for continuous variables or as numbers and percentages for categorical variables. Univariate Cox's proportional-hazards regression model was used to assess hazard ratios (HR) and 95% confidence intervals (CI) of TB occurrence between each treatment and control group. To achieve a balance of patient's age, sex, and disease duration between groups in IBD patients, a one-to-one greedy matching (one case per one control) with the nearest-neighbor algorithm was performed. The same matching procedure was conducted within CD and UC patients. The incidence of TB and its 95% CI for each treatment group was calculated per 10,000 person-years. The median days on medication before TB was defined as the median value of the medication period from the initial to the onset of TB in each treatment group. A stratified Cox proportional-hazards regression analysis for the matched-pair data were used to evaluate the relative hazard of events in the treatment group compared to the control group. Cumulative incidence for the study outcome was estimated using the Kaplan-Meier method. A two-sided *P*-value < 0.05 was considered to be statistically significant. All statistical analyses were performed using the SAS Enterprise Guide software version 6.1 (SAS Institute Inc., Cary, NC, USA).

## 3 Results

### 3.1 IBD incidence in South Korea

**Figure 2:**
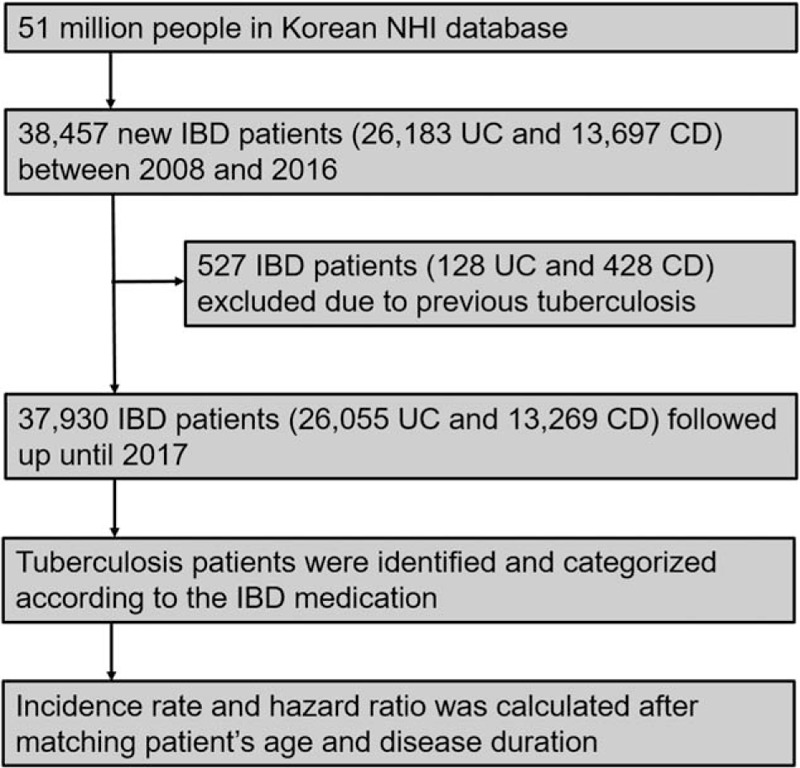
Flowchart of the study.

Between 2008 and 2015, a total of 38,457 patients were newly diagnosed as IBD (26,183 as UC and 13,697 as CD) (Fig. 2). The average annual incidence of IBD per 100,000 persons during 2008 and 2015 was 9.9, 6.5 for UC and 3.4 for CD, and the incidences were stable without major changes throughout the study period. The average UC-to-CD incidence ratio was 1.9. After excluding 128 patients with UC and 428 patients with CD for previous TB history, 26,055 patients with UC and 13,269 patients with CD were followed until December 2016.

The baseline characteristics of the study population are presented in Table 1. Males showed higher incidences in both UC and CD. The percentage of male IBD patients were 61.9%, 58.4% in the UC group and 68.9% in the CD groups. The mean diagnostic age for IBD patients were 38.9 years, 43.2 years for UC and 30.6 years for CD. It is notable that the mean diagnostic age of CD patients was younger than that of UC patients. The highest incidence of UC was found in aged 40–59 years of old while it was 20-39 years of old for CD patients.

**Table 1:**
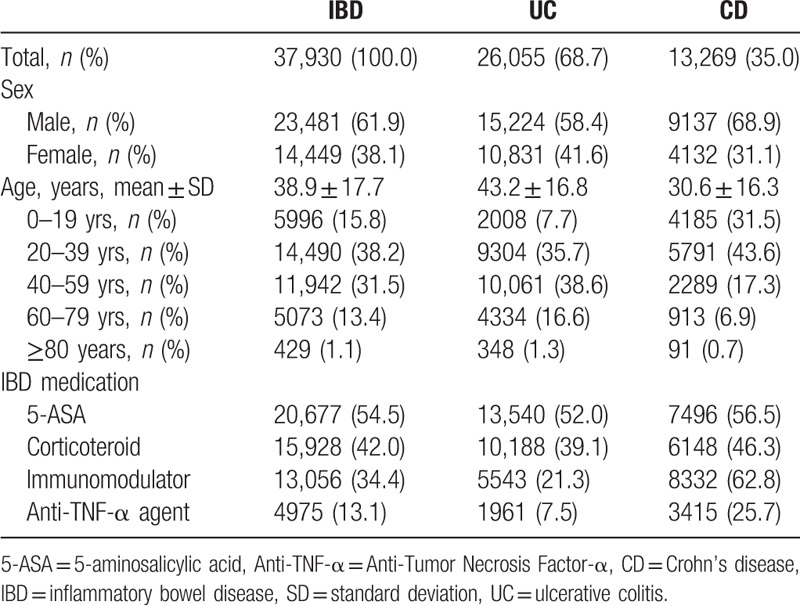
Baseline characteristics of inflammatory bowel disease patients.

### 3.2 Risk of TB in IBD patients according to the medication

**Figure 3:**
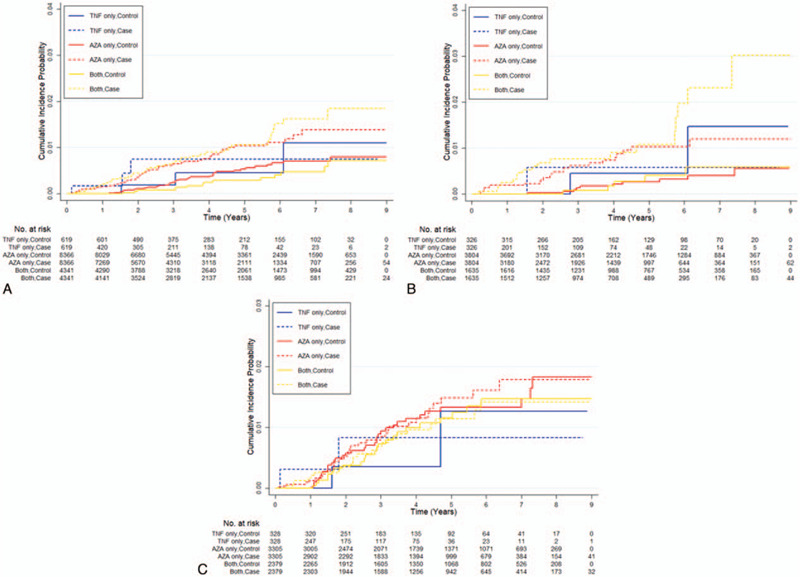
Comparison of tuberculosis incidence according to drug. (A) In IBD patients, the cumulative incidence of TB was significantly elevated in the AZA group and more in the combination group. (B) The results were similar in the UC group. (C) This pattern was, however, not observed in the CD group. AZA = azathioprine only, CD = Crohn's disease, IBD = inflammatory bowel disease, TB = tuberculosis, UC = ulcerative colitis.

In IBD patients, a total of 249 patients were identified as active TB patients. After one-to-one matching with patient's age, sex, and disease duration, the hazard of TB incidence were significantly higher in CAI group (HR, 5.67; 95% CI, 2.42–10.21, *P* < .001) than WI group, followed by AISS group (HR, 3.50; 95% CI, 1.92–6.37, *P* < .001), AIM group (HR, 3.26; 95% CI, 1.18–9.05, *P* < .001), and AZA group (HR, 2.06; 95% CI, 1.35–3.12, *P* < .001) (Table 2). Figure 3 shows the cumulative incidence probability of TB in the group of IBD (left), UC (middle), and CD (right). The median days on IBD medication before onset of TB were WI, 1074 days for WI group, 569 days for IFX group, 764 days for AZA group, and 319 days for combination groups.

**Table 2:**
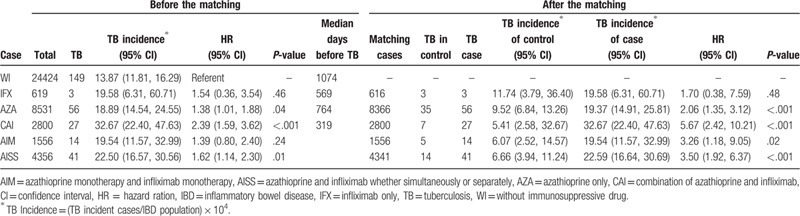
Tuberculosis risk in inflammatory bowel disease before and after matching.

### 3.3 Risk of TB in UC patients according to medication

In UC patients, a total of 135 patients were identified as active TB patients. When variables of age, sex, and disease duration were matched, the hazard of TB was the highest in AISS group (HR, 4.84; 95% CI, 1.80–13.0, *P* < .001), followed by CAI group (HR, 4.50; 95% CI, 1.71–11.82, *P* < .001), and by AZA group (HR, 3.33; 95% CI, 1.63–6.82, *P* < .001), all compared with WI group. However, the hazard of TB in IFX group was not significantly differ from WI group (HR, 1.18; 95% CI, 0.13–10.86) (Table 3). The median days on IBD medication before onset of TB for WI group, IFX group, AZA group, and combination groups were 1181 days, 569 days, 809 days, and 296 days, respectively.

**Table 3:**
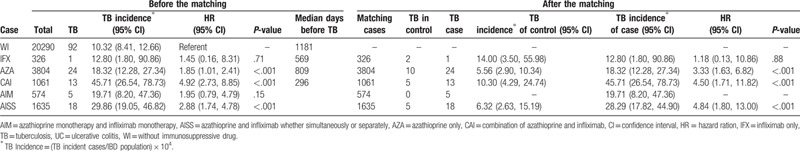
Tuberculosis risk in ulcerative colitis before and after matching.

### 3.4 Risk of TB in CD patients according to medication

In CD patients, a total of 131 patients were identified as active TB patients. After matching, the hazards of TB for all of IFX, AZA, CAI, AIM, and AISS groups were not significantly differ from that of WI group (Table 4). Before onset of TB, the median days on IBD medication were as follows: WI, 979 days; IFX, 355 days, AZA, 778 days; and combination groups, 323 days.

**Table 4:**
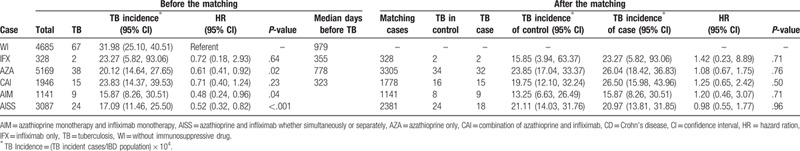
Tuberculosis risk in Crohn's disease before and after matching.

## 4 Discussion

Only few population studies have previously reported the risk of incident TB in patients with IBD; however, our study further analyzed the long-term risk of TB in IBD patients according to IBD treatment regimen including combination therapy in a large-scale cohort.^[16,17]^ It is difficult to estimate the risk of TB in IBD patients through clinical trials owing to the low incidence of both IBD and TB; thus, the results might be unreliable. Our nationwide study demonstrated in large IBD population that the risk of TB increase significantly after treatment using azathioprine, infliximab and especially their combination treatment.

We calculated the HR of TB in each IBD treatment regimen and performed matching analysis with the confounding factors of age, sex, and disease duration, which may affect the HR and can be retrieved from our data source. In matched data in IBD, the HR of TB increased significantly in the following order: AZA (HR = 2.06, *P* = <.001), AIM (HR = 3.26, *P* = .02), AISS (HR = 3.50, *P* = <.001), and CAI (HR = 5.67, *P* = <.001), suggesting that azathioprine increases TB in IBD patients and its combination with infliximab increases the risk more despite having the shortest median days on medication before TB. This pattern is also shown in UC patients; however, our data could not prove that azathioprine, infliximab, nor their combination increases the TB risk in CD patients. These patterns are similar in cumulative incidence probability of TB and can be visually seen in Figure 3.

The results from such big data should be carefully evaluated and interpreted. Our results using this big data have a few notable points that are different from common knowledge. First, our results show that the HR of TB increases in the CD group in general, whereas no increase was observed with immunomodulators or biologics. However, it is widely accepted that these drugs increase TB risk regardless of minor variations in the underlying disease.^[18,19]^ We interpreted this result to a limitation in using big data, which lacks important clinical or endoscopic data. Because of their phenotypic similarities, CD can be mistaken for intestinal TB (ITB), and vice versa. ITB is a differential diagnosis of CD that is often misdiagnosed as TB, as one literature reported the ratio of misdiagnosis to be 10.8%.^[20]^ When we identified TB regardless of TB medication, the TB incidence of WI group was much higher than expected, and 62% of TB in CD patients were diagnosed in intestine suggesting that some of ITB patients were included in the CD group. As we apply more strict definition of TB to remove the possible ITB from CD patients, some of real CD patients were removed inevitably from the case. Second, although elevated, HR of TB from IFX group failed to show significance. However in our study, IFX group does not truly represent all patients who received infliximab therapy because AIM group also includes the patients with infliximab monotherapy. It can be assumed that because HR of AIM (3.26 in IBD) is higher than HR of AZA (2.06 in IBD), infliximab increases the risk of TB; however, it is difficult to distinguish the true effect of each drugs in AIM group.

The risk of TB in treatment against biologics and immunomodulators has been studied thoroughly by many researchers.^[10,16–19,21]^ TNF, a cytokine produced by CD4 T cell, plays a pivotal role in cellular immunity.^[22]^ Anti-TNF therapy increases the risk of reactivation of infectious granulomatous pathogens such as TB by interfering with innate and adaptive immunity in many different ways.^[21]^ Azathioprine exerts its immunosuppressive action by affecting lymphocyte-mediated cellular immunity with a reduction in macrophage activation and inhibition of lymphocyte activation.^[23]^ Because of these pharmacologic entities, azathioprine has been used in the treatment of not only in IBD but also rheumatologic diseases, organ transplantation, and hematologic malignancies. Combination therapy of anti-TNF agent and immunomodulators have supported the fact that the therapeutic effect would be amplified by different target mechanisms, and a recent study suggested that the effect of combination therapy is owing to reduced immunogenicity and elevated infliximab blood concentration.^[24]^ Hence, we assumed that combination therapy has synergistic risks in TB as compared with monotherapy using each drug. The results of our study show that the HR of CAI (5.67 in IBD) is higher than the total HR of IFX and AZA (1.70 and 2.06 in IBD, respectively), and is also higher than the HR of AIM (3.26 in IBD), thus suggesting that TB risk increases when these drugs are used in combination in a synergistic fashion. Interestingly, a higher risk of TB was found in UC patients in AZA group when compared to those in IFX group (HR 3.33 vs 1.18). Because most patients who receive anti-TNF treatment are previously exposed to immunomodulators, this data is rare to obtain. Higher incidence of TB was expected in UC patients who were treated with Anti-TNF agent compared to immunomodulators, but our result was the opposite. Due to the small number even in nationwide registry-based analysis, the result must be interpreted carefully, and more studies are needed to support the result.

One of the strengths of our study is that variables used in the study represent their group well. We chose infliximab for analysis among anti-TNF agent because not only is it the first and mostly widely used anti-TNF agent for IBD, but also it is a chimeric IgG monoclonal antibody that shows a higher incidence of TB compared with soluble TNF receptors.^[25]^ Azathioprine is also the first and most widely used immunodulator for IBD. Because these two agents were the first in their categories, they are suitable for long-term follow-up studies. TB might well be regarded as the representative of an immune-mediated disease because of its high cumulative incidence, and South Korea is a country with intermediate TB burden, which causes IBD patients to become more exposed to TB.^[26,27]^ However, further studies in different settings are needed to generalize our results.

The present study had several limitations. First, the nature of the retrospective cohort study design could result in ascertainment bias, and there is a limitation in the nationwide insurance database itself. Because the database was collected for the purpose of reimbursement, it could be inaccurate and limited for analysis. For instance, there were patients with diagnostic codes of both UC and CD (3%), and it was impossible to find out what the correct diagnosis was based on these data alone. In addition, important clinical data like IBD disease activity or severity were missing in the data, hence their influences were neglected. We used matching analysis to overcome the limitation of the database and reduce the confounders. Moreover, several small event cases may not be enough for generalization despite statistical significance. Because IBD and TB are both diseases with considerably low incidence rates, we used the nationwide database to overcome this limitation; however, TB incidence among IBD patients remain limited. Small numerical changes could bring about large outcome differences, hence a careful interpretation is required, and well-designed prospective randomized controlled study is needed to support the results.

## 5 Conclusions

In conclusion, our nationwide population-based study showed that combination therapy of infliximab and azathioprine in IBD patients showed an increased HR of TB and a considerably higher HR than those obtained by monotherapy. This result suggests that further intensive screening and surveillance are required before and after the IBD treatment and especially when using immunomodulators and/or biologics.

## Author contributions

**Conceptualization:** Seong Ji Choi, Eun Sun Kim.

**Formal analysis:** Jae Min Lee, Hyuk Soon Choi.

**Investigation:** Bora Keum, Yoon Tae Jeen.

**Methodology:** Min Sun Kim, Jae Min Lee.

**Supervision:** Eun Sun Kim, Jae Min Lee, Chang Duck Kim.

**Writing – original draft:** Seong Ji Choi, Min Sun Kim.

**Writing – review & editing:** Hong Sik Lee, Hoon Jai Chun.

All authors have read and agreed to the final version of the manuscript.
